# Insecticide Susceptibilities and Enzyme Activities of Four Stink Bug Populations in Mississippi, USA

**DOI:** 10.3390/insects15040265

**Published:** 2024-04-12

**Authors:** Yuzhe Du, Shane Scheibener, Yu-Cheng Zhu, K. Clint Allen, Gadi V. P. Reddy

**Affiliations:** 1 Southern Insect Management Research Unit, Agriculture Research Service, United States Department of Agriculture, 141 Experiment Station Road, Stoneville, MS 38776, USA; shane.scheibener@usda.gov (S.S.); clint.allen@usda.gov (K.C.A.); gadi.reddy@usda.gov (G.V.P.R.); 2 Pollinator Health in Southern Crop Ecosystems Research Unit, Agriculture Research Service, United States Department of Agriculture, 141 Experiment Station Road, Stoneville, MS 38776, USA; yc.zhu@usda.gov

**Keywords:** Pentatomidae complex, insecticide susceptibility, acephate, pyrethroids and neonicotinoids, spray bioassay, detoxification enzymes, baseline

## Abstract

**Simple Summary:**

In Mississippi, the stink bug complex infesting soybean is mainly composed of the brown stink bug, southern green stink bug, green stink bug, and redbanded stink bug. This study conducted spray bioassays to assess the susceptibilities of these stink bugs to seven commonly used formulated insecticides. Stinks bugs were collected from soybeans in Leland, MS, USA, at the Southern Insect Management Research Unit (SIMRU) Farm during 2022 and 2023, as well as from wild host plants in Clarksdale, MS. While the green stink bug exhibited consistent susceptibility across two years, the redbanded stink bug showed slightly increased susceptibility to neonicotinoids in 2023 compared to 2022. In 2022, the susceptibility ranking among species was redbanded stink bug ≤ green stink bug ≈ southern green stink bug, whereas in 2023, it was redbanded stink bug ≤ green stink bug ≤ brown stink bug. Enzyme activity analysis revealed no significant differences between the southern green and the green stink bug. However, compared to SIMRU-2023, populations of brown stink bug and redbanded stink bug from SIMRU-2022 and Clasksdale-2023 exhibited elevated enzyme activities, indicating potential variations in resistance mechanisms. These findings provide valuable insights for monitoring and managing insecticide resistance in Mississippi soybean.

**Abstract:**

In Mississippi, the Pentatomidae complex infesting soybean is primarily composed of *Euschistus servus*, *Nezara viridula*, *Chinavia hilaris*, and *Piezodorus guildinii*. This study employed spray bioassays to evaluate the susceptibilities of these stink bugs to seven commonly used formulated insecticides: oxamyl, acephate, bifenthrin, λ-cyhalothrin, imidacloprid, thiamethoxam, and sulfoxaflor. Stinks bugs were collected from soybeans in Leland, MS, USA during 2022 and 2023, as well as from wild host plants in Clarksdale, MS. There was no significant difference in the susceptibility of *C. hilaris* to seven insecticides between two years, whereas *P. guildinii* showed slightly increased susceptibility to neonicotinoids in 2023. Among all four stink bug species, susceptibility in 2022 was ranked as *P. guildinii* ≤ *C. hilaris* ≈ *N. viridula*, while in 2023, it was ranked as *P. guildinii* ≤ *C. hilaris* ≤ *E. Servus*. Additionally, populations of *E. servus* and *P. guildinii* collected from Clarksdale exhibited high tolerance to pyrethroids and neonicotinoids. Moreover, populations of *E. servus* and *P. guildinii* from SIMRU-2022 and Clarksdale-2023 showed elevated esterase and cytochrome P450 activity, respectively. These findings from spray bioassays and enzyme activity analyses provide a baseline for monitoring insecticide resistance in Pentatomidae and can guide insecticide resistance management strategies for Mississippi soybean.

## 1. Introduction

Stink bugs (Pentatomidae) are polyphagous insects that feed on various agricultural crops, including cotton, soybeans, and maize, and they are economically significant in the southeastern United States [1,2,3]. In Mississippi, the Pentatomidae complex infesting soybeans (*Glycine max* (L.) Merr.) typically consists of the southern green stink bug (*Nezara viridula* (Linnaeus)), green stink bug (*Chinavia hilaris* (Say)), brown stink bug (*Euschistus servus* (Say)), and redbanded stink bug (*Piezodorus guildinii* (Westwood)) [1,2,4,5]. Among these stink bugs, *C. hilaris* (previously *Acrosternum hilare* (Say)) and *E. servus* are considered two of the most damaging native stink bug species in the United States. *Nezara viridula* originated from Ethiopia and has now spread worldwide [1,2], while *P. guildinii* originated from the Caribbean basin and invaded the southern U.S. states [1,2]. Between 1987 and 1991, *P. guildinii* accounted for approximately 20% of the total stink bugs collected on maturity group VIII soybean in Georgia [6]. Stink bugs damage soybeans by inserting their stylets through the pods into the seed and injecting salivary secretions that facilitate ingestion, which causes shriveled, discolored, deformed seeds, and sometimes, complete loss of pods [7]. This injury not only alters the nutritional value of soybean seeds but also creates entry points for plant pathogens [2]. Damage caused by stink bugs, particularly *P. guildinii*, can also lead to leaf retention, a condition that delays soybean plant maturation by maintaining the green leaves even after pod maturation [8,9]. 

Managing Pentatomidae pests poses significant challenges as they can quickly reach high infestation levels and cause severe seed damage, often requiring chemical control in cotton and soybean-producing states in the mid-south and southeastern regions [10,11,12]. Currently, insecticide applications for stink bug control often include neonicotinoids, organophosphates, and pyrethroids. For instance, imidacloprid, thiamethoxam, and a commercially available mixture of λ-cyhalothrin and thiamethoxam are commonly used to control *E. servus* [4,5]. However, legislation limiting insecticide use and the evolution of resistance in the target pests can significantly impact insecticide efficacy [11,12]. For example, until 2010, acephate was the primary product recommended for controlling *P. guildinii* in Louisiana [10]. Concerns about resistance development arose due to the reliance on a single product, as multiple applications of acephate per season were necessary to control this pest [10]. Despite some stink bug species developing resistance to certain insecticides, synthetic insecticides remain the primary method for Pentatomidae control [10,11,12,13,14].

The mechanism underlying resistance to insecticides in Pentatomidae can be attributed to multiple factors, including target-site mutations, increased metabolism, and behavioral changes. However, to date, no description of target-site mutations has been reported in the Pentatomidae complex. Three groups of enzymes play critical roles in the mechanisms of insecticides resistance: cytochrome P450 complex (P450), glutathione S-transferases (GST), and esterase. Esterase represents a broad group of isoenzymes with multifunctional hydrolytic activities, catalyzing the hydrolysis of numerous ester bonds, and thus, conferring resistance to a broad range of insecticides [15]. GSTs catalyze the secondary metabolism of a vast array of compounds oxidized by the cytochrome P450 families, which are multifunctional enzymes implicated in detoxifying insecticides in insects [16]. Resistance to insecticides containing ester bonds may occur through metabolic detoxification via hydrolysis and subsequent sequestration [17]. Cytochrome P450s are well known for their role in insecticide resistance, as insects use these enzymes to metabolize various xenobiotic substrates, converting them from a toxic insoluble form to water-soluble forms [18,19,20]. Studying esterase, GST, and cytochrome P450 patterns and identifying enzymes involved in the insecticide detoxification in insect pests can enhance our understanding of resistance mechanisms to these compounds and contribute to pest control programs.

Despite the development of resistance in stink bugs due to increased insecticide applications rates, a challenge exists in rearing stink bugs in the laboratory to obtain susceptible populations, which is critical for establishing baseline insecticide mortality data. Such data were obtained for *E. servus*, *C. hilaris*, and *N. viridula* nearly 20 years ago in Mississippi [11]. Since then, newer pesticides have been formulated and new stink bugs, such as *P. guildinii*, have been introduced. Hence, this study aims to reevaluate the susceptibilities of these stink bugs, incorporating *P. guildinii*. Spray bioassays were conducted to determine the LC_50_ values of four stink bug species to seven commonly used formulated insecticides, including oxamyl, acephate, bifenthrin, λ-cyhalothrin, imidacloprid, thiamethoxam, and sulfoxaflor. The Pentatomidae complex was collected from soybeans near Leland, MS, USA, at the Southern Insect Management Research Unit (SIMRU) Farm during the fall of 2022 and 2023. Additionally, another stink bug population was collected from wild host plants in Clarksdale, Coahoma County, north Mississippi in October 2023. This study also assessed the three detoxification enzymes and acetylcholinesterase (AChE) activity levels in these four species. Therefore, this study not only determined the baseline levels of susceptibility to currently commonly used insecticides through spray bioassays but also assessed the levels of esterase, GST, AChE, and cytochrome P450 activities in the populations of *N. viridula*, *C. hilaris*, *E. servus*, and *P. guildinii*, for two consecutive years in two locations in Mississippi.

## 2. Materials and Methods

### 2.1. Insect Collections

The populations of brown stink bug (*Euschistus servus* (Say)), southern green stink bug (*Nezara viridula* (L.)), green stink bug (*Chinavia hilaris* (Say)), and redbanded stink bug (*Piezodorus guildinii* (Westwood)) adults were obtained late-season from soybean (*Glycine max* (L.)) fields at the Southern Insect Management Research Unit (SIMRU) Farm (N 33°20′51.7″, W 90°54′55.1″), located 8 km south of Leland, Mississippi, during September and October 2022 and 2023. Additionally, other populations of *E. servus*, *C. hilaris*, and *P. guildinii* were collected from wild host plants in October 2023 from Clarksdale (N 34°11′39.306″, W 90°28′0.156″), Coahoma County, Mississippi, an area with a long history of cotton, soybean, and maize production and frequent insecticide uses for insect pest management [21]. The insects were collected using a modified leaf blower with a sweep net and held in a polypropylene cage (12′ × 12′ × 12′) overnight before setting up bioassays the following day. 

### 2.2. Insecticides

The insecticides used in this study were formulated insecticides, including Warrior II (λ-cyhalothrin, 22.8%, Syngenta, Greensboro, NC, USA), Tundra ^®^EC (bifenthrin, 25.1%, Winfield Solution LLC, St. Paul, MN, USA), Bracket (acephate, 97%, Winfield Solution LLC), Vydate C-LV (Oxamyl, 42%, Dupont, Wilmington, DE, USA), Advise^®^ 2FL (imidacloprid, 40.4%, Winfield Solutions LLC), Centric 40WG (thiamethoxam, 40%, Syngenta), and Transform 5G (Sulfoxaflor, 50%, Syngenta) (Supplementary Table S1). These insecticides were purchased from local agricultural chemical suppliers near Stoneville, Mississippi, and stored in a refrigerator at 4 ± 1 °C.

### 2.3. Laboratory Spray Bioassays Containing Bean Pods (Phaseolus vulgaris L.)

Field-collected *E. servus*, *C. hilaris*, *N. viridula*, and *P. guildinii* adults were placed in plastic cups (500 mL round wide-mouth polypropylene cup; DxH: 9.3 × 10 cm; n = 8/cup for *E. servus*, *C. hilaris*, and *N. viridula*, and 20/cup for *P. guildinii*) containing fabric mesh-covered holes (5.0 cm in diameter) cut into both the lid and bottom. The procedure for the spray bioassays was similar to that described previously [22]. The stink bug adults were considered dead if they were unable to walk or fly. Each treatment was replicated 3 or 4 times (for each insecticide concentration) with 40 *E. servus*, *C. hilaris*, and *N. viridula*, and 100 *P. guildinii* adults per replicate. Five concentrations of each insecticide were used to determine the LC_50_ values and 95% confidence intervals from the three or four replicates. 

### 2.4. Detoxification Enzyme Activity Assays 

#### 2.4.1. Chemicals

For the enzyme activity assays, the following chemicals were purchased from Sigma-Aldrich (St. Louis, MO, USA): protease inhibitor (cocktail tablets), α-naphthyl acetate, α-naphthol, sodium lauryl sulphate, fast blue B salt, 1-chloro-2,4-dinitrobenzene (CDNB), L-glutathione-reduced (GSH), acetylthiocholine (ATC), 5,5-dithiobis 2-nitrobenzoic acid (DTNB), umbelliferone (7-hydroxycoumarin), 7-ethoxycoumarin (7-EC), acetonitrile, and Trizma base buffer.

#### 2.4.2. Enzyme Preparation

Adult *E. servus*, *C. hilaris*, *N. viridula* (n = 1/rep, head and thorax), and *P. guildinii* (n = 2/rep, head and thorax) were homogenized (Homogenizer, Thomas Scientific, Swedesboro, NJ, USA) in 500 μL of ice-cold sodium phosphate buffer (0.1 M, pH 7.2) containing 0.1% Triton X-100 and a protease inhibitor in a 2.0 mL screw cap tube. The homogenates were centrifuged at 10,000× *g* for 15 min at 4 °C, and the resulting supernatant was transferred to a 1.5 mL centrifuge tube. An undiluted sample was used for the cytochrome P450 assay, a 4-fold dilution of supernatant was used for the protein, GST, and AChE assays, and a 20-fold dilution was used for the esterase assay. The supernatants were diluted using homogenization buffer without Triton X-100. The total protein concentration of each enzyme extract was determined using a Bradford protein assay kit with a bovine serum albumin standard [23] (Thermo Scientific, Waltham, MA, USA). Each sample for all assays was measured with three technical replicates.

#### 2.4.3. Esterase Activity Assays

Esterase activity against α-naphthyl acetate was determined by adapting the assay methods of Zhu et al. and Dorman et al. [24,25]. Briefly, 10 μL of enzyme solution (diluted in 0.1 M sodium phosphate buffer, pH 7.2) and 135 μL of 0.3 mM α-naphthyl acetate solution were added to each well. The reaction solution was incubated at 37 °C for 30 min, and the reaction was stopped by adding 50 μL fast blue B salt (3 mg/mL) to 5% sodium lauryl sulfate solution. After a 15 min incubation period at room temperature, the absorbance values were measured at 600 nm using a Biotek Synergy H1 plate reader (Agilent, Winooski, VT, USA). The esterase activity was calculated based on the standard linear relationship established using α-naphthol per minute per milligram of protein. 

#### 2.4.4. Glutathione S-Transferase (GST) Activity Assays

Glutathione S-transferase (GST) activity was determined using CDNB as the substrate, following the modified protocols of Zhu et al. [24]. The reaction mixture (120 μL) consisted of 10 μL of the enzyme solution, 10 μL of 2 mM CDNB, 50 μL of 10 mM GSH, and 50 μL of 0.1 M sodium phosphate buffer (0.1 M, pH 7.5). The optical density at 340 nm (OD340) was continuously measured every 10 s for a total of 10 min using a Biotek Synergy H1 plate reader. The GST activity was determined based on the extinction coefficient of 5.3 mM^−1^ cm^−1^ for CDNB and reported as nmol/min/mg [26].

#### 2.4.5. Acetylcholinesterase (AChE) Activity Assay

Acetylcholinesterase (AChE) activity was measured using acetylthiocholine (ATC) according to the method of Zhu et al. [24], with some modifications. Each reaction mixture included 50 μL enzyme extract, 0.25 mM ATC, and 0.4 mM DTNB in 150 μL of 0.1 M phosphate buffer pH 7.5. The enzyme activity, expressed as Vmax mOD/min, was determined kinetically at 405 nm using a Biotek Synergy H1 plate reader. The AChE activity was expressed as nmol ATC hydrolyzed per min per mg protein using the extinction coefficient of 1.36 × 10^4^ M^−1^ cm^−1^.

#### 2.4.6. Cytochrome P450 Monooxygenase (P450) Assays

Cytochrome P450 activity was quantified by a CYP450-mediated deethlylation of 7-exthoxycoumarin (7-EC) to 7-hydroxycoumarin reaction according to the protocols of Zhu et al. and Dorman et al. [24,25], with some modifications. In each well (black 96-well flat-bottom microplate), 40 µL of the enzyme solution was mixed with 76 μL of sodium phosphate buffer (0.1 M, pH 7.2) and 4 μL of 8 mM 7-EC in 95% ethanol as a substrate. The plate was incubated at 37 °C (200 rpm) for 4 hrs in an incubator shaker (Thermo Scientific, Waltham, MA, USA). The reaction was stopped with 120 μL 50% (*v*/*v*) acetonitrile in 50 mM TRIZMA-base buffer (pH = 10). The fluorescence of 7-hydroxycoumarin was measured with a Biotek Synergy H1 plate reader at 460 nm while exciting at 360 nm. The cytochrome P450 activity (7-EC-O-deethylation, ECOD) was determined based on the 7-hydroxycoumarin standard curve, and the protein concentration and activity were expressed as the 7-hydroxycoumarin formed per minute per milligram of protein.

### 2.5. Data Analysis

For the bioassay data, the lethal concentration 50 (LC_50_) values and 95% confidence intervals were calculated by Probit analysis using SPSS software (version 19.0, SPSS Inc., Chicago, IL, USA, 2003), and the data are presented as the means ± S.D. If there was no overlap in the 95% confidence intervals, the LC_50_ values of different populations were considered significantly different. Student’s *t*-test was applied to determine the statistically significant differences in mortality vs. 50%. The esterase, GST, cytochrome P450, and AChE enzymatic activities were plotted using JMP 17.0 software. A one-way analysis of variance followed by Tukey’s HSD was used to determine statistically significant differences (*p* < 0.05).

## 3. Results

### 3.1. Spray Bioassay in SIMRU-2022

In the 2022 lab spray bioassay, only abundant populations of *N. viridula*, *C. hilaris*, and *P. guildinii* were included, as *E. servus* was insufficient for the bioassay. No mortality from distilled water was observed in any bioassays for any of the four stink bug species tested. *Chinavia hilaris* treated with oxamyl and acephate showed LC_50_ values ranging from 65.60 to 116.27 µg/mL and from 125.03 to 200.95 µg/mL, respectively (Table 1). The responses of *N. viridula* adults to oxamyl and acephate were slightly higher but not significantly different, with LC_50_ values ranging from 113.79 to 168.32 µg/mL and from 183.40 to 244.95 µg/mL, respectively. *Piezodorus guildinii* exhibited a similar susceptibility to oxamyl as *N. viridula* and *C. hilaris* but was more susceptible to acephate than *C. hilaris* (3.3-fold) and *N. viridula* (4.4-fold). The LC_50_ values for *C. hilaris* exposed to the pyrethroid insecticides of bifenthrin and λ-cyhalothrin ranged from 3.84 to 7.92 µg/mL and from 2.64 to 9.99 µg/mL, respectively (Table 1). While the susceptibilities to λ-cyhalothrin and bifenthrin were slightly variable, they were not significantly different in three of the stink bug species. *Nezara viridula*, *P. guildinii*, and *C. hilaris* adults showed similar responses to both pyrethroids. Regarding the neonicotinoid insecticides, the LC_50_ values for *C. hilaris* adults ranged from 15.05 to 31.31 µg/mL for imidacloprid and from 8.62 to 19.18 µg/mL for thiamethoxam (Table 1). *Nezara viridula* was more tolerant to imidacloprid than *C. hilaris* and *P. guildinii*, by about 3.4- and 4.3-fold, respectively, while the responses to thiamethoxam were not significantly different among the three of them. Additionally, the LC_50_ values for *C. hilaris* adults exposed to sulfoxaflor ranged from 129.55 to 227.03 µg/mL (Table 1), showing similar effects to *N. viridula*, but they had 4.3-fold diminished susceptibility compared to *P. guildinii.*

### 3.2. Spray Bioassay in SIMRU-2023

In the 2023 spray bioassay, the stink bug populations changed, including only abundant *E. servus*, *C. hilaris*, and *P. guildinii* from soybeans at the SIMRU Farm. The *N. viridula* population was insufficient for bioassays. The LC_50_ values in 2023 ranged from 73.57 to 131.60 µg/mL and from 73.70 to 265.43 µg/mL for oxamyl and acephate in *C. hilaris*, respectively (Table 2). While *E. servus* adults exhibited 3.6-fold lower susceptibility to oxamyl, there was no significant difference in their susceptibility to acephate compared with *C. hilaris*. Conversely, *P. guildinii* adults were 3.8-fold more susceptible to acephate, but their responses to oxamyl were not significantly different. The LC_50_ values for *C. hilaris* exposed to bifenthrin and λ-cyhalothrin ranged from 6.85 to 11.60 µg/mL and from 4.46 to 10.68 µg/mL, respectively (Table 2). While *C. hilaris* and *P. guildinii* adults showed no significant difference, *E. servus* adults displayed significantly (3.4- and 6.3-fold) lower susceptibility to bifenthrin and λ-cyhalothrin, respectively, compared to *C. hilaris*. The LC_50_ values for *C. hilaris* adults exposed to neonicotinoid insecticides ranged from 17.31 to 31.61 µg/mL and from 8.01 to 17.01 µg/mL for imidacloprid and thiamethoxam, respectively (Table 2). The population of *E. servus* was 5.9- and 7.7-fold more tolerant to imidacloprid and thiamethoxam, respectively. In contrast, *P. guildinii* was 2.7- and 2.4-fold more susceptible to imidacloprid and thiamethoxam, respectively, than *C. hilaris* (Table 2). The LC_50_ values for *C. hilaris* adults exposed to sulfoxaflor were 276.09 µg/mL (Table 2), which was 1.5-fold less than those for *E. servus* and 4.5-fold more than those for *P. guildinii*.

The sensitivities of the seven formulated pesticides were examined in both *C. hilaris* and *P. guildinii* between two consecutive years, 2022 and 2023. *Chinavia hilaris* exhibited no significant difference in susceptibility (overlap of 95% confidence intervals) to all seven pesticides. In contrast, *P. guildinii* showed 2.1- and 2.2-fold more susceptibility to imidacloprid and thiamethoxam in 2023 than in 2022, while the susceptibility to the other five insecticides was similar in both years (Table 1 and Table 2, Supplementary Table S2).

### 3.3. Spray Bioassay in Clarksdale-2023 

**Figure 1:**
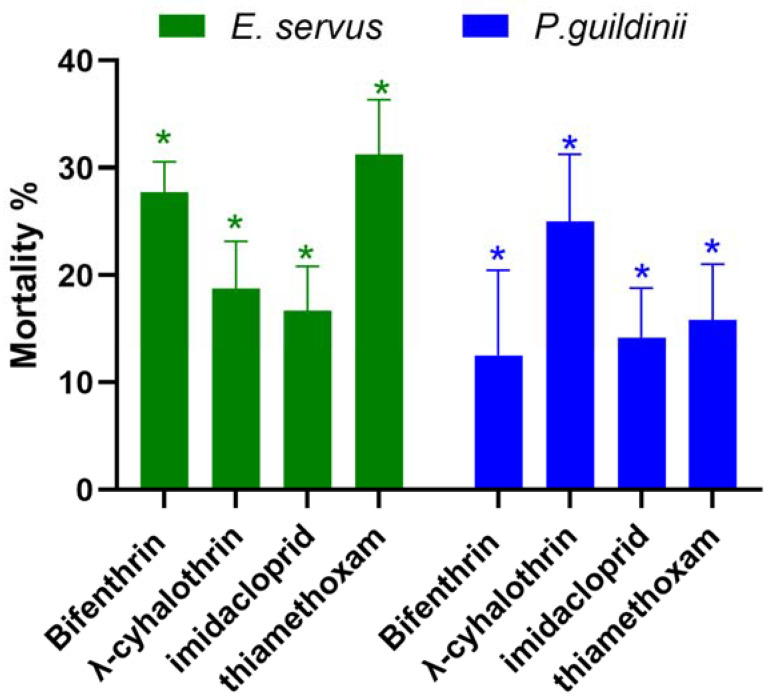
Mortality (%) of the populations of Clarksdale-2023 brown stink bug (*Euschistus servus*) and redbanded stink bug (*Piezodorus guildinii*) following treatment with LC_50_ concentrations of bifenthrin, λ-cyhalothrin, imidacloprid, and thiamethoxam in SIMRU-2023. The concentrations used were 30, 30, 140, and 95 µg/mL for *E. servus*, and 10, 10, 10, and 10 ug/mL for *P. guildinii*, respectively. The asterisks denote statistically significant differences in mortality compared to 50%, as determined by Student’s *t*-test (*p* < 0.05).

We collected *P. guildinii* and *E. servus* from wild host plants in Clarksdale, North Mississippi, where the stink bug populations were not sufficient for five concentrations to determine the LC_50_ value. Therefore, *P. guildinii* and *E. servus* from the Clarksdale-2023 population were exposed to single concentrations of four pesticides at around or higher than the LC_50_ values calculated from the SIMRU-2023 population to assess differences in their susceptibility. Bifenthrin, λ-cyhalothrin, imidacloprid, and thiamethoxam were applied at concentrations of 10, 10, 10, and 10 µg/mL, and 30, 30, 140, and 95 µg/mL to *P. guildinii* and *E. servus*, respectively (Table 2). The Clarksdale-2023 population of *P. guildinii* and *E. servus* adults had significantly lower mortalities when exposed to LC_50_ concentrations of both pyrethroids and neonicotinoids from the SIMRU Farm applications (Figure 1). Across the four pesticides, mortality ranged from 16.7 to 31.2% in *E. servus* and 12.5 to 25.0% in *P. guildinii* compared to the 50% mortality (Figure 1). 

### 3.4. Detoxification Enzymes and AChE Activity Assays

**Figure 2:**
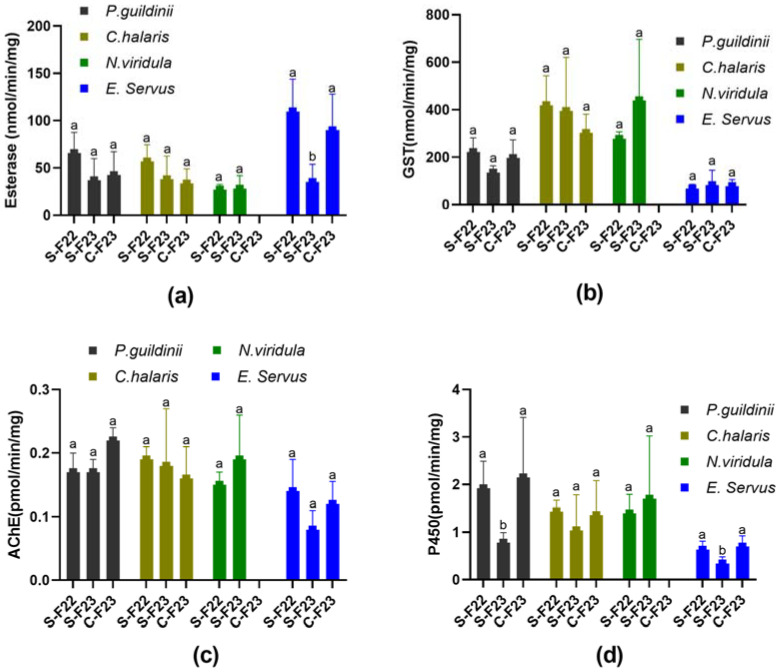
The enzyme activities of esterase (**a**), glutathione S-transferases (GST) (**b**), acetylcholinesterase (AChE) (**c**), and cytochrome P450 complex (P450) (**d**), for green stink bug (*Chinavia halaris*), southern green stink bug (*Nezara Viridula*), brown stink bug (*Euschistus servus*), and redbanded stink bug (*Piezodorus guildinii*) from three different populations: SIMRU-2022 (S-F22), SIMRU-2023 (S-F23), and Clarksdale-2023 (C-F23). Statistically significant differences were identified within each group of enzymes. Means sharing different letters on the top of the bars are significantly different, as determined using one-way analysis of variance with Tukey’s HSD test, and significant values were set at *p* < 0.05.

The esterase, GST, and cytochrome P450 activities were assessed in the populations of *E. servus*, *N. viridula*, *C. hilaris*, and *P. guildinii* collected from SIMRU Farm in 2022 and 2023 (SIMRU-2022, SIMRU-2023), as well as from Clarksdale in 2023 (Clarksdale-2023). In SIMRU-2022, *C. hilaris* exhibited esterase, GST, AChE, and cytochrome P450 activities of 58.86 nmol/min/mg, 419.03 nmol/min/mg, 0.19 pmol/min/mg, and 1.44 pmol/min/mg, respectively (Supplementary Table S3). However, SIMRU-2023 and Clarksdale-2023 showed no significant differences in esterase, GST, AChE, or cytochrome P450 activities (Figure 2). Similarly, *N. viridula* displayed esterase, GST, AChE, and cytochrome P450 activities of 27.20 nmol/min/mg, 276.68 nmol/min/mg, 0.15 pmol/min/mg, and 1.40 pmol/min/mg, respectively, in SIMRU-2022 (Figure 2, Supplementary Table S3), with no significant differences compared to SIMRU-2023 (Figure 2). No population of *N. viridula* was available from Clarksdale-2023. For *P. guildinii*, the esterase, GST, AChE, and cytochrome P450 activities were 65.71 nmol/min/mg, 221.47 nmol/min/mg, 0.17 pmol/min/mg, and 1.92 pmol/min/mg, respectively, in SIMRU-2022 (Supplementary Table S3). No significant differences in esterase, GST, or AChE activities were observed among three *P. guildinii* populations, whereas SIMRU-2023 exhibited significantly lower (2.5- and 2.7-fold) cytochrome P450 activities compared to SIMRU-2022 and Clarksdale-2023, respectively (Figure 2). *Euschistus servus* displayed esterase, GST, AChE, and cytochrome P450 activities in SIMRU-2022 of 109.83 nmol/min/mg, 62.76 nmol/min/mg, 0.14 pmol/min/mg, and 0.63 pmol/min/mg, respectively (Supplementary Table S3). There was no significant difference in the GST or AChE activities among the three populations. However, the populations of SIMRU-2023 *E. servus* showed significantly lower (3.1- and 2.6-fold) esterase, and 1.9- and 2.1-fold diminished cytochrome P450 activities compared to SIMRU-2022 and Clarksdale-2023, respectively (Figure 2). 

## 4. Discussion

Stink bug adults were collected from the soybean fields at the SIMRU Farm in Leland, MS, USA, over two consecutive years (2022 and 2023). The spray bioassay results indicate that the susceptibility of *C. hilaris* to all seven insecticides tested was very similar in 2022 and 2023, whereas *P. guildinii* was slightly more susceptible to two neonicotinoids in 2023 compared to 2022. However, susceptibilities can vary between stink bug species and their life stages [11,27,28]. In 2022, *N. viridula* and *C. hilaris* showed similar susceptibility to six insecticides, with *N. viridula* displaying greater tolerance to imidacloprid. In contrast, in 2023, *E. servus* demonstrated reduced susceptibilities to six insecticides evaluated, excluding acephate, compared to *C. hilaris*. These findings are consistent with previous studies conducted twenty years ago, which reported that *E. servus* exhibited less susceptibility to pyrethroid insecticides compared to *N. viridula* and *C. hilaris* [11]. In the current study, *E. servus* exhibited 3.4-fold and 6.3-fold reduced susceptibilities to bifenthrin and λ-cyhalothrin, respectively, compared to *N. viridula*. Snodgrass et al. had similar findings in Mississippi (2- to 10-fold higher LC_50_) for bifenthrin and cyhalothrin when comparing the same species (*E. servus* and *N. viridula*) [11]. However, *E. servus* was more challenging to control with products recommended for *N. viridula* in Louisiana. Insecticide efficacy trials in soybean fields indicated that λ-cyhalothrin LC_50_ for *E. servus* adults was 51 times higher than that of *N. viridula* adults [28]. Meanwhile, compared to *C. hilaris* and *N. viridula*, *E. servus* has similar susceptibility to acephate in this study, with lower susceptibility twenty years ago [11,28]. At that time, organophosphates such as acephate and pyrethroids were the only products recommended for controlling stink bugs, and reliance on these single products for control raised the selection pressure [10]. Moreover, our results show that *N. viridula*, *C. hilaris*, and *P. guildinii* were similarly susceptible to two pyrethroids. *P. guildinii* was more susceptible to acephate in both years. However, previous bioassay results in Louisiana differed; *P. guildinii* showed 4- to 8-fold and 2- to 8-fold less susceptibility to pyrethroids and organophosphates, respectively, compared to *N. viridula* [14]. These differences suggest potential resistance development and the introduction of a new insecticide. For example, due to the fast development of resistance, pyrethroids were only recommended for use at certain times for insect pests such as *Lygus lineolaris* (Palisot de Beauvois), which could have influenced resistance patterns in recent years [29]. Additionally, *E. servus* also exhibited diminished susceptibility to neonicotinoids, while *P. guildinii* adults were more susceptible to neonicotinoids than *N. viridula* and *C. hilaris*. These differences in susceptibility can be attributed to variations in pesticide application, leading to species-specific responses. Some stink bugs, such as *N. viridula* and *P. guildinii*, are distributed worldwide. In Brazil, the neotropical brown stink bug (*Euschistus heros* (Fabricius)), from the same genus as *E. servus*, showed diminished susceptibility to various neonicotinoid and pyrethroid formulated mixtures, whereas *N. viridula* and *P. guildinii* were most susceptible to the insecticides evaluated, which is similar to results in the US [30].

The spray tower bioassay conducted in this study provided valuable data on the susceptibility of stink bugs to various insecticides. However, comparing the LC_50_ value directly with previous studies can be challenging due to variations in susceptibility based on the active ingredient and deployment method [31,32]. Various bioassay techniques, including topical and glass vial bioassays, dipping bioassays, and ingestion methods, each have their advantages and limitations, whereas application methods may not fully account for differences in susceptibility. The choice of spray bioassay in this study was appropriate, considering its relevance to field conditions where insecticide sprays are commonly used for pest control. Topical and vial bioassays are also frequently used methods for lab bioassays [10,11,30]. Glass-vial bioassays can determine the LC_50_ value, while topical application bioassays and tarsal contact bioassays can be conducted using the vial test, allowing for direct contact between insects and insecticides [11]. Dipping bioassays involve exposing insects to insecticides by dipping fresh green bean pods in a solution containing the insecticide [33]. More recently, novel ingestion methods have been used to monitor stink bug susceptibility to insecticides [34]. Hence, a standardized test for measuring insecticide resistance in stink bugs should be created.

Additionally, compared to the populations from the SIMRU Farm, populations of *E. servus* and *P. guildinii* collected from wild host plants in Clarksdale, MS exhibited higher tolerance to two pyrethroid and neonicotinoid insecticides. However, this increased tolerance does not necessarily indicate resistance in the field against the doses used by the farmers. The populations from SIMRU were directly collected from soybean fields, with fewer pesticide treatments, whereas Clarksdale, located in north Mississippi, Coahoma County, has a long history of cotton, maize, and soybean production, with frequent insecticide use for insect pest management. Stink bug populations were collected at the end of October 2023 after harvest, a period when susceptibility may be lower compared to the beginning of the season [22]. The frequent use of the same mode of action of insecticides may favor the selection of resistant populations, possibly contributing to resistance in Clarksdale-2023 stink bugs. Stink bug management is often challenged by the rapid development of insecticide resistance, leading to increased insecticide applications and the use of broad-spectrum products [10,14,35]. Insecticide resistance also poses a significant challenge in pest management and agriculture, as it diminishes the effectiveness of insecticides, resulting in control failure. This occurs when insecticides fail to achieve the desired level of pest control due to various factors, including insecticide resistance, improper application techniques, inadequate dosage, environmental factors, and incomplete coverage of the target area [36]. Stink bug populations significantly impact soybean yields and insecticide resistance cases have been reported in many countries. In South America, the main species impairing soybean yields include *N. viridula*, *P. guildinii*, and *E. heros*. Failure to control *E. heros* populations has been reported in Brazil for β-cyfluthrin, bifenthrin, λ-cyhalothrin, and imidacloprid [34,37].

Detoxifying enzyme activity assays revealed that the Clarksdale-2023 population of *P. guildinii* exhibited significantly higher cytochrome P450 activity levels, along with increased tolerance to pyrethroids and neonicotinoids. Similarly, the Clarksdale-2023 population of *E. servus* showed reduced susceptibility to these insecticides, coupled with significantly elevated esterase and cytochrome P450 activities. Although the bioassay for the SIMRU-2022 *E. servus* population was unavailable, it demonstrated higher esterase and cytochrome P450 activities compared to the SIMRU-2023 population. Therefore, the reduced susceptibilities to neonicotinoids and pyrethroids in both the *P. guildinii* and *E. servus* populations are associated with a potential metabolic resistance mechanism involving elevated esterase and cytochrome P450 activities. Our results are consistent with those of *E. heros* in Brazil; analyses of the activities of several enzymes (α-esterase, β-esterase, GST, AChE, and P450) in *E. heros* showed that the highest activities were detected in populations with well-known low susceptibility to insecticides [37], and two imidacloprid-resistant *E. heros* strains exhibited higher cytochrome P450 activity by 72.3% and 40.5% [38]. In addition, our findings establish a baseline of four enzyme activity profiles across four stink bug species. However, the esterase activity in *P. guildinii* was lower than that observed in the Louisiana (Brazil) population, where esterase activity ranged from 251 to 658 nmol α-naphthol formed/min/mg protein. Esterase activity levels in Londrina (Brazil) populations averaged 163 nmol/min/mg [10], possibly due to variations in enzyme preparation procedures [10]. Moreover, assaying with synergistic agents, such as enzyme inhibitors, in future studies may further reveal which detoxification enzymes play a major role in detoxifying different insecticides in field populations of each stink bug species.

Stink bug management in soybean cultivation currently relies on chemical control methods, including neonicotinoids, pyrethroids, and organophosphates. However, growing concerns about pesticide resistance in stink bugs are prompting pest managers to explore safer and more sustainable options [39]. Biological control strategies involving parasitoids and entomopathogens, as well as the utilization of semiochemicals (including sex pheromones), are being employed [40]. Moreover, biological control through the use of egg and adult parasitoids and the development of cultivars tolerant to stink bug damage are being explored. For instance, the well-studied egg parasitoid *Trissolcus basalis* (Wollaston) has been successfully used by farmers in Brazil [41].

## 5. Conclusions

This study provides additional baseline data on the current tolerance levels of *E. servus*, *N. viridula*, *C. hilaris*, and *P. guildinii* to seven common insecticides using three populations at two locations during two years in Mississippi. There was no significant difference in the susceptibility of *C. hilaris* to insecticides between 2022 and 2023, whereas *P. guildinii* was slightly more susceptible to neonicotinoids in 2023 than in 2022. Among all four stink bug species, populations of *N. viridula* and *C. hilaris* had similar susceptibilities to the insecticides evaluated except for imidacloprid; *E. servus* exhibited diminished susceptibility to six insecticides, while *P. guildinii* displayed higher susceptibility to acephate and the tested neonicotinoids. Moreover, the populations of *E. servus* and *P. guildinii* collected from Clarksdale, MS, exhibited higher tolerance to two pyrethroids/neonicotinoids. Additionally, the activity levels of three detoxification enzymes and AChE in the four stink bug species were evaluated. The results confirm that the neonicotinoid- and pyrethroid-resistant populations of *E. servus* and *P. guildinii* were associated with a potential metabolic resistance mechanism involving elevated esterase and cytochrome P450 activity levels. This study not only establishes baseline levels of susceptibility to seven commonly used insecticides but also provides insights into the activity levels of detoxification enzymes. The data from these tests are valuable for future studies on resistance and for resistance monitoring programs for stink bugs.

## Figures and Tables

**Table 1:** Spray bioassay results of seven formulated insecticides against the adult green stink bug (*Chinavia halaris*), southern green stink bug (*Nezara viridula*), and redbanded stink bug (*Piezodorus guildinii*) 48 h post-treatment from SIMRU Farm, Leland, MS, USA, in 2022.

Compounds ^1^	Population	Slope	LC_50_ (μg/mL) ^2^	95% Confidence Intervals (μg/mL) ^2^	χ^2^	*p*
Oxamyl	*C. halaris*	2.391 ± 0.395	88.84 ^a^	65.60–116.27	0.29	0.99
	*N. viridula*	3.028 ± 0.464	140.10 ^a^	113.79–168.32	1.37	0.85
	*P. guildinii*	3.221 ± 0.331	97.55 ^a^	85.69–117.46	6.52	0.16
Acephate	*C. halaris*	2.842 ± 0.485	157.88 ^a^	125.03–200.95	2.07	0.56
	*N. viridula*	4.783 ± 0.817	213.36 ^a^	183.40–244.66	1.59	0.45
	*P. guildinii*	2.811 ± 0.327	48.36 ^b^	40.61–55.80	2.54	0.28
Bifenthrin	*C. halaris*	2.011 ± 0.379	5.68 ^a^	3.84–7.92	2.35	0.50
	*N. viridula*	2.163 ± 0.383	5.57 ^a^	3.86–7.45	3.09	0.38
	*P. guildinii*	1.944 ± 0.301	8.40 ^a^	6.66–10.53	2.80	0.25
λ-Cyhalothrin	*C. halaris*	1.339 ± 0.343	6.13 ^a^	2.64–9.99	0.70	0.95
	*N. viridula*	2.168 ± 0.598	3.14 ^a^	1.37–4.56	0.82	0.66
	*P. guildinii*	1.378 ± 0.200	5.97 ^a^	4.13–7.78	0.43	0.93
Imidacloprid	*C. halaris*	2.065 ± 0.405	22.17 ^b^	15.05–31.31	1.53	0.68
	*N. viridula*	2.074 ± 0.364	74.58 ^a^	57.04–103.23	3.84	0.28
	*P. guildinii*	1.770 ± 0.222	17.39 ^b^	13.74–21.24	3.78	0.29
Thiamethoxam	*C. halaris*	2.077 ± 0.534	13.67 ^a^	8.62–19.18	1.40	0.50
	*N. viridula*	1.768 ± 0.366	15.22 ^a^	10.12–20.81	0.28	0.96
	*P. guildinii*	1.688 ± 0.233	11.45 ^a^	8.57–14.23	0.40	0.94
Sulfoxaflor	*C. halaris*	2.711 ± 0.545	174.04 ^a^	129.55–227.03	2.44	0.49
	*N. viridula*	2.663 ± 0.422	223.62 ^a^	180.86–287.57	3.43	0.49
	*P. guildinii*	1.272 ± 0.263	90.52 ^b^	66.84–128.10	0.88	0.83

^1^ All the compounds were formulated pesticides. ^2^ LC_50_ values and 95% confidence intervals were calculated by Probit analyses using SPSS software. Letters ^a^ and ^b^ indicate significant differences with no overlap in the 95% confidence intervals between different stink bug populations.

**Table 2:** Spray bioassay results of seven formulated insecticides against the adult green stink bug (*Chinavia halaris*), brown stink bug (*Euschistus servus*), and redbanded stink bug (*Piezodorus guildinii)* 48 h post-treatment from SIMRU Farm, Leland, MS, USA, in 2023.

Compounds ^1^	Population	Slope	LC_50_ (μg/mL) ^2^	95% Confidence Intervals (μg/mL) ^2^	χ^2^	*p*
Oxamyl	*C. halaris*	2.085 ± 0.491	98.89 ^b^	73.57–131.60	1.12	0.29
	*E. servus*	1.801 ± 0.612	356.56 ^a^	237.91–714.94	2.41	0.30
	*P. guildinii*	3.109 ± 0.447	87.97 ^b^	73.20–102.35	0.12	0.73
Acephate	*C. halaris*	2.400 ± 0.973	163.21 ^a^	73.70–256.43	2.07	0.56
	*E. servus*	4.958 ± 1.405	269.96 ^a^	209.31–312.61	1.31	0.25
	*P. guildinii*	3.668 ± 0.531	43.51 ^b^	36.54–50.10	0.41	0.52
Bifenthrin	*C. halaris*	2.121 ± 0.411	9.07 ^b^	6.85–11.60	1.85	0.17
	*E. servus*	2.980 ± 0.512	31.07 ^a^	23.91–39.74	0.47	0.79
	*P. guildinii*	0.897 ± 0.312	10.11 ^b^	5.61–18.79	0.03	0.85
λ-Cyhalothrin	*C. halaris*	1.343 ± 0.289	7.22 ^b^	4.46–10.68	0.01	0.99
	*E. servus*	1.972 ± 0.425	45.28 ^a^	32.54–67.52	0.14	0.93
	*P. guildinii*	2.360 ± 0.465	7.58 ^b^	5.37–9.49	0.07	0.80
Imidacloprid	*C. halaris*	3.783 ± 0.905	23.77 ^b^	17.31–31.61	0.11	0.74
	*E. servus*	1.763 ± 0.444	139.53 ^a^	93.57–329.16	1.47	0.48
	*P. guildinii*	1.281 ± 0.237	8.87 ^c^	5.50–11.40	0.43	0.51
Thiamethoxam	*C. halaris*	1.567 ± 0.278	12.22 ^b^	8.01–17.01	1.82	0.34
	*E. servus*	2.084 ± 0.500	94.36 ^a^	68.16–139.57	0.43	0.81
	*P. guildinii*	1.191 ± 0.211	5.18 ^c^	2.90–7.55	0.67	0.72
Sulfoxaflor	*C. halaris*	6.666 ± 1.762	276.09 ^b^	231.43–320.99	0.12	0.73
	*E. servus*	5.638 ± 2.191	425.08 ^a^	363.36–607.45	1.51	0.22
	*P. guildinii*	0.998 ± 0.234	61.95 ^c^	37.78–86.06	1.80	0.41

^1^ All the compounds were formulated pesticides. ^2^ LC_50_ values and 95% confidence intervals were calculated by Probit analyses using SPSS software. Letters ^a^, ^b^, and ^c^ indicate significant differences with no overlap in the 95% confidence intervals between different stink bug populations.

## Data Availability

All the data are reported in the manuscript. Additional details are available from the corresponding author upon request.

## Supplementary Materials

The following supporting information can be downloaded at: https://www.mdpi.com/article/10.3390/insects15040265/s1. Table S1: The commercial name, common name (with percentage active ingredient), manufacturer, mode of action (MOA) for insecticides and rates used on stink bug spp; Table S2: Spray bioassay results (LC_90_) of seven formulated insecticides against the adult green stink bug, *Chinavia halaris*; southern green stink bug, *Nezara Viridula*; brown stink bug, *Euschistus Servus*; red banded stink bug, *Piezodorus guildinii* 48 h post-treatment from SIMRU Farm, Leland, MS. during 2022 and 2023; Table S3: The enzyme activities of esterase, glutathione S-transferases (GST), cytochrome P450 complex (P450) and acetylcholinesterase (AChE) in green stink bug, *Chinavia halaris*; southern green stink bug, *Nezara Viridula*; brown stink bug, *Euschistus servus* and redbanded stink bug, *Piezodorus guildinii* from three different populations: SIMRU-2022, SIMRU-2023 and Clarksdale-2023.
