# Supplementary material for: Insecticide Susceptibilities and Enzyme Activities of Four Stink Bug Populations in Mississippi, USA
Source: Insects. 2024 Apr 12;15(4):265. doi: 10.3390/insects15040265 (PMC11050663; doi:10.3390/insects15040265)
Supplement: Supplementary file 1 [file insects-15-00265-s001.zip › insects-2935373-supplementary.pdf]

Table S1. The commercial name, common name (with percentage active ingredient), manufacturer, mode of action (MOA) for insecticides and rates used on stink bug spp.

	Commercial Name	Common Name (% a. i)	Manufacturer	Mode of Action	Amount of formulation per Acre	Pounds Active Ingredient per Acre
1	Vydate C-LV	Oxamyl (42%)	Dupont	1A	1.5-4 pt	0.24-0.64
2	Bracket	Acephate (97%)	Winfield Solution	1B	0.056-1.1 lb	0.056-1.1
3	Tundra®EC	Bifenthrin (25.1%)	Winfield Solution	3A	2.1-6.4 oz	0.033-0.1
4	Warrior II	λ-cyhalothrin (22.8%)	Syngenta	3A	1.60-2.56 oz	0.025-0.04
5	Advise®2FL	Imidacloprid (40.4%)	Winfield Solution	4A	2.0-3.0 oz	0.02-0.04
6	Centric 40WG	Thiamethoxam (40%)	Syngenta	4A	2.0-2.5 oz	0.02-0.03
7	Transform	Sulfoxaflor (50%)	Syngenta	4C	0.75-2.75 oz	0.023-0.086

Table S2. Spray bioassay results (LC_{90}) of seven formulated insecticides against the adult green stink bug, *Chinavia halaris*; southern green stink bug, *Nezara Viridula*; brown stink bug, *Euschistus Servus*; red banded stink bug, *Piezodorus guildinii* 48 hours post-treatment from SIMRU Farm, Leland, MS. during 22022 and 2023.

Compounds[1]	Population SIMRU-2022	LC_{90} (μg/mL)[2]	95% Confidence Intervals (μg/mL)[2]	Population SIMRU-2023	LC_{90} (μg/mL)[2]	95% Confidence Intervals (μg/mL)[2]
Oxamyl	*C. halaris*	305.3 [a]	213.7– 562.8	*C. halaris*	347.9 [b]	158.9– 1312.8
	N. viridula	371.2[a]	287.6 – 567.2	*E. servus*	1835.6[a]	836.6 – 4308.2
	P. guildinii	243.8 [a]	207.0 – 305.9	*P. guildinii*	227.3 [b]	183.2 – 321.5
Acephate	*C. halaris*	445.9 [a]	319.7 – 814.9	*C. halaris*	558.1 [a]	315.8 – 1537.1
	N. viridula	395.4 [a]	328.9 – 547.4	*E. servus*	489.6 [a]	397.6 – 920.7
	P. guildinii	138.2 [b]	114.3 – 181.6	*P. guildinii*	97.28 [b]	80.80 – 130.4
Bifenthrin	*C. halaris*	24.63[a]	15.71 – 57.86	*C. halaris*	27.48 [a]	15.19 – 112.6
	N. viridula	21.81 [a]	14.92 – 42.91	*E. servus*	83.63 [a]	60.99 – 145.4
	P. guildinii	38.32 [a]	25.80 – 76.93	*P. guildinii*	27.21[a]	15.80 – 98.45
λ-Cyhalothrin	*C. halaris*	55.51 [a]	20.16 – 153.7	*C. halaris*	36.37 [b]	12.64 – 89.39
	N. viridula	17.99 [a]	10.71 – 81.70	*E. servus*	204.20 [a]	98.51 – 479.4
	P. guildinii	50.83 [a]	33.68 – 101.0	*P. guildinii*	26.47 [b]	19.25 – 50.12
Imidacloprid	*C. halaris*	92.53 [b]	58.11 – 231.8	*C. halaris*	51.86 [b]	37.52 – 107.7
	N. viridula	309.41 [a]	191.6 – 775.3	*E. servus*	591.6 [a]	274.9 – 1692.4
	P. guildinii	92.08 [b]	66.26 – 153.15	*P. guildinii*	47.27[b]	29.20 – 96.43
Thiamethoxam	*C. halaris*	56.57[a]	34.36 –217.0	*C. halaris*	79.56[b]	57.81 –175.5
	N. viridula	80.81 [a]	49.49 – 231.2	*E. servus*	296.6 [a]	183.4 – 983.4
	P. guildinii	65.76 [a]	47.13 – 112.5	*P. guildinii*	61.66 [b]	35.70 – 171.8
Sulfoxaflor	*C. halaris*	516.8 [a]	360.2 – 1074.0	*C. halaris*	429.8 [a]	358.6 – 695.0
	N. viridula	677.1 [a]	472.8 – 1278.4	*E. servus*	717.4 [a]	542.3 – 2338.8
	P. guildinii	307.1 [a]	193.9 – 821.1	*P. guildinii*	180.9 [b]	102.4 – 326.3

[1] All the compounds were formulated pesticides.

[2] LC_{50} values and 95% confidence intervals were calculated by Probit analyses using SPSS software. Letters a and b indicate significant difference with no overlap in the 95% confidence intervals between different stink bug populations.

Table S3. The enzyme activities of esterase, glutathione S-transferases (GST), cytochrome p450 complex (P450) and acetylcholinesterase (AChE) in green stink bug, *Chinavia halaris*; southern green stink bug, *Nezara Viridula*; brown stink bug, *Euschistus servus* and red banded stink bug, *Piezodorus guildinii* from three different populations: SIMRU-2022, SIMRU-2023 and Clarksdale-2023.

Species	Population	Esterase (nmol/min/mg)	GST (μmol/min/mg)	AChE (pmol/min/mg)	P450 (pmol/min/mg)
C. halaris	SIMRU-2022	56.86 ± 3.55	419.03 ± 24.73	0.19 ± 0.02	1.44 ± 0.25
	SIMRU-2023	38.16 ± 4.87	395.25 ± 44.97	0.18 ± 0.02	1.04 ± 0.13
	Clarksdale-2023	33.64 ± 3.08	302.24 ± 15.73	0.16 ± 0.01	1.36 ± 0.13
N. viridula	SIMRU-2022	27.20 ± 2.37	276.68 ± 13.56	0.15 ± 0.01	1.40 ± 0.15
	SIMRU-2023	28.14 ± 2.75	439.88 ± 51.26	0.19 ± 0.01	1.70 ± 0.26
P. guildinii	SIMRU-2022	65.71 ± 4.38	221.47 ± 12.08	0.17 ± 0.01	1.92 ± 0.10 [a]
	SIMRU-2023	37.09 ± 4.56	134.69 ± 5.78	0.17 ± 0.00	0.78 ± 0.04 [b]
	Clarksdale-2023	42.40 ± 4.97	218.65 ± 13.36	0.22 ± 0.02	2.15 ± 0.27 [a]
E. servus	SIMRU-2022	109.83 ± 6.81 [a]	62.76 ± 3.32	0.14 ± 0.01	0.63 ± 0.03 [a]
	SIMRU-2023	35.19 ± 3.75 [b]	67.49 ± 3.68	0.08 ± 0.01	0.34 ± 0.02 [b]
	Clarksdale-2023	89.96 ± 12.08 [a]	78.27 ± 8.61	0.12 ± 0.01	0.70 ± 0.14 [a]

Statistically significant differences were identified within each group of enzymes, sharing different letter are significantly different, as determined using one-way analysis of variance with Tukey's HSD test, and significant values were set at $p < 0.05$.
